# The antihypertensive drug hydralazine activates the intrinsic pathway of apoptosis and causes DNA damage in leukemic T cells

**DOI:** 10.18632/oncotarget.7871

**Published:** 2016-03-03

**Authors:** María J. Ruiz-Magaña, Rocío Martínez-Aguilar, Estefanía Lucendo, Diana Campillo-Davo, Klaus Schulze-Osthoff, Carmen Ruiz-Ruiz

**Affiliations:** ^1^ Unidad de Inmunología, IBIMER, Universidad de Granada, Granada, Spain; ^2^ Interfaculty Institute of Biochemistry, University of Tübingen, Tübingen, Germany; ^3^ German Cancer Consortium (DKTK) and German Research Cancer Center (DKFZ), Heidelberg, Germany; ^4^ Departamento de Bioquímica y Biología Molecular 3 e Inmunología, Facultad de Medicina, Universidad de Granada, Granada, Spain

**Keywords:** hydralazine, apoptosis, mitochondria, DNA damage, leukemia

## Abstract

Epigenetic therapies have emerged as promising anticancer approaches, since epigenetic modifications play a major role in tumor initiation and progression. Hydralazine, an approved vasodilator and antihypertensive drug, has been recently shown to act as a DNA methylation inhibitor. Even though hydralazine is already tested in clinical cancer trials, its mechanism of antitumor action remains undefined. Here, we show that hydralazine induced caspase-dependent apoptotic cell death in human p53-mutant leukemic T cells. Moreover, we demonstrate that hydralazine triggered the mitochondrial pathway of apoptosis by inducing Bak activation and loss of the mitochondrial membrane potential. Hydralazine treatment further resulted in the accumulation of reactive oxygen species, whereas a superoxide dismutase mimetic inhibited hydralazine-induced cell death. Interestingly, caspase-9-deficient Jurkat cells or Bcl-2- and Bcl-x_L_-overexpressing cells were strongly resistant to hydralazine treatment, thereby demonstrating the dependence of hydralazine-induced apoptosis on the mitochondrial death pathway. Furthermore, we demonstrate that hydralazine treatment triggered DNA damage which might contribute to its antitumor effect.

## INTRODUCTION

Three main epigenetic alterations, DNA methylation, histone modifications and non-coding RNAs, affect gene expression and play a central role in many types of diseases, including cancer development and progression. In particular, silencing of tumor suppressor genes by promoter hypermethylation has been described in a variety of human solid tumors such as lung, colorectal, breast, gastric, endometrial and bladder tumors, as well as in hematopoietic malignancies [1-3], contributing to clonal expansion of malignant cells.

Interestingly, epigenetic modifications can be reversed by pharmacological agents, such as DNA methylation inhibitors, which suggest the valuable therapeutic potential of these type of drugs in cancer [4]. Conventional demethylating agents include the nucleoside analogues 5-azacytidine and decitabine. Several reports have confirmed that treatment with these nucleosides analogues causes the up-regulation of apoptosis- and cell cycle-associated genes, and induces cell death in tumor cells, thus demonstrating their antitumor effect [5-8]. Decitabine and 5-azacytidine are being currently used to treat myelodysplastic syndrome and have been recently approved for the treatment of acute myeloid leukemia in Europe. In addition, phase I clinical trials with decitabine, either alone or in combination with other drugs such as histone deacetylase inhibitors, have been conducted in patients with refractory malignancies and solid tumors [9]. The main problems associated with these nucleoside analogues are their poor stability and high toxicity. Zebularine, a chemically stable cytidine analogue, exhibits demethylating activity together with low toxicity in both in vivo and in vitro studies [10, 11]. During the last years, a group of non-nucleoside analogue compounds with different structures has emerged as new demethylating agents that prevent the toxic effects derived from the incorporation into DNA. The antiarrhythmic drug procainamide, the antihypertensive drug hydralazine and the green tea polyphenol (−)-epigallocatechin-3-gallate, among others, belong to this group [5, 12].

Hydralazine is a potent vasodilator that has been widely used to treat hypertension during pregnancy as well as heart failure [13, 14]. Recently, it has been shown to act as a DNA methylation inhibitor by reducing the expression of the DNA methyltransferases DNMT1 and DNMT3a, which, together with DNMT3b, are the enzymes responsible for cytosine methylation in mammals [15, 16]. In addition, binding models have been developed that support a high affinity interaction between hydralazine and DNMT1 [15, 17]. In comparative studies, hydralazine as well as other non-nucleoside DNTM inhibitors have shown lower ability to inhibit DNA methylation and reactivate methylation-silenced genes in tumor cells than conventional nucleoside inhibitors [18]. Nonetheless, hydralazine is currently under evaluation in clinical trials for cancer therapy, mainly in combination with a histone deacetylase inhibitor [19-21], even though there is limited understanding of its precise mechanism of action.

T-cell acute lymphoblastic leukemia is a type of cancer with a high frequency of mutations in genes encoding for epigenetic regulators, which suggests a therapeutic potential of epigenetic drugs for the treatment of this hematologic malignancy [22]. We have recently described the induction of apoptosis by the nucleosides analogues decitabine and zebularine in leukemic T cells [23]. In the present study, we have evaluated the ability of hydralazine to induce cell death in this hematologic malignancy. Our results indicate that this DNTM inhibitor triggers caspase-dependent apoptosis in p53-mutant leukemic T cells. Similarly to decitabine and zebularine, we have found that hydralazine activates the mitochondrial apoptotic pathway and induces DNA damage.

## RESULTS

### Hydralazine induces caspase-dependent apoptosis in leukemic T cells

**Figure 1:**
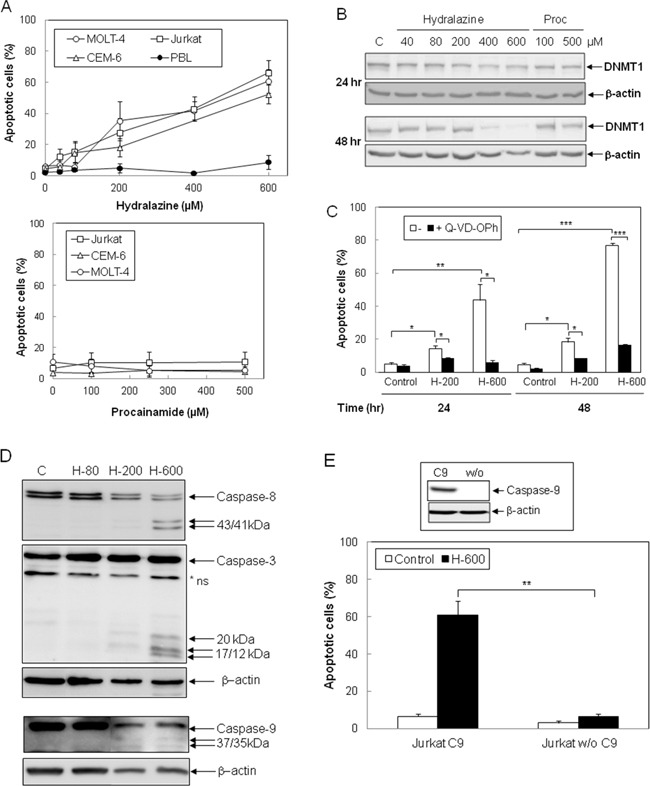
Induction of caspase-dependent apoptosis by hydralazine in leukemic T cells **A.** Jurkat, CEM-6, MOLT-4 and peripheral blood lymphocytes (PBL) were treated with different doses of hydralazine (40, 80, 200, 400 and 600 μM; upper panel) or procainamide (100, 250 and 500 μM; lower panel) for either 48 hr (T cell lines) or 72 hr (PBL in upper panel). **B.** Expression of DNTM1 was determined by Western blotting in Jurkat cells after treatment for 24 and 48 hr without (C) or with the indicated concentrations of hydralazine and procainamide. **C.** Jurkat cells were preincubated for 1 hr in the absence or in the presence of the caspase inhibitor Q-VD-OPh (20 μM) before treatment without (control) or with 200 and 600 μM hydralazine (H-200 and H-600, respectively) for 24 and 48 hr. **D.** Activation of caspases-8, -3 and -9 was determined by Western blot in Jurkat cells treated without (C) or with 80, 200 and 600 μM hydralazine (H-80, H-200 and H-600, respectively) for 48 hr. β-Actin was used as loading control. **E.** Caspase-9-deficient (Jurkat w/o C9) and caspase-9-reconstituted (Jurkat C9) Jurkat cells were treated without (control) or with 600 μM hydralazine (H-600) for 48 hr. The inset figure shows the levels of caspase-9 and β-actin in caspase-9-reconstituted and -lacking cells, as determined by Western-blot. A nonspecific protein was detected by the caspase-3 antibody (*ns). In A, C and E, sub-G1 apoptotic cells were analyzed by flow cytometry. Error bars show SEM from three independent experiments. *p < 0.05; **p < 0.01; ***p < 0.001.

To evaluate whether hydralazine induces apoptosis in leukemic T cells, three different cell lines, Jurkat, MOLT-4 and CEM-6, were incubated with hydralazine in a range of doses previously reported to reduce DNTM expression and activity [24, 25]. As shown in Figure 1A, hydralazine induced apoptosis in a dose-dependent manner in all leukemic T cell lines studied. In contrast, procainamide, another non-nucleoside analogue DNMT inhibitor, did not induce apoptosis in leukemic T cells (Figure 1A, lower panel). Significant apoptosis was observed in Jurkat and MOLT-4 cells at concentrations of 200 μM and above as soon as 24 hr after treatment with hydralazine, while CEM-6 cells appeared slightly less sensitive (Figure 1A, 1C and data not shown). Interestingly, peripheral blood lymphocytes from healthy donors were highly resistant to hydralazine, even when incubated for three days with the higher dose of 600 μM (Figure 1A, upper panel). We verified the ability of hydralazine and procainamide to inhibit DNMTs by analyzing the expression of DNMT1. As shown in Figure 1B, high doses of hydralazine induced a slight decrease in DNMT1 protein expression at 24 hr and nearly a complete loss after 48 hr of treatment, whereas procainamide had almost no effect on DNMT1 expression.

Next, we studied the involvement of caspases in cell death induced by hydralazine. The pan-caspase inhibitor Q-VD-OPh completely inhibited hydralazine-mediated apoptosis (Figure 1C). Moreover, proteolytic processing of the initiator caspases-8 and -9, as well as cleavage of the effector caspase-3, was observed upon treatment with 200 and 600 μM hydralazine (Figure 1D). To further confirm the role of caspases in the induction of apoptosis by hydralazine, we studied the response of a caspase-9-deficient Jurkat clone to this demethylating agent [23, 26]. As shown in Figure 1E, caspase-9-deficient Jurkat cells (Jurkat w/o C9) were completely resistant to hydralazine-induced apoptosis, in contrast to deficient cells stably transfected with a caspase-9 expression vector (Jurkat C9).

### The intrinsic apoptotic pathway is triggered by hydralazine in leukemic T cells

**Figure 2:**
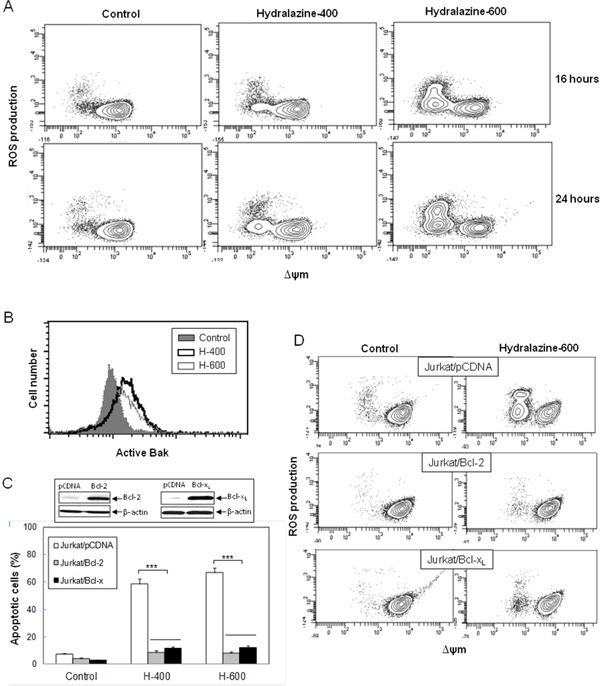
Hydralazine activates mitochondrial apoptotic events in leukemic T cells **A.** Jurkat cells were treated without (control) or with 400 and 600 μM hydralazine for 16 and 24 hr. Mitochondrial membrane potential (ΔΨ_m_) and ROS production were determined by flow cytometry using DIOC_6_(3) and dihydroethidium, respectively. **B.** Bak activation was analyzed by flow cytometry in Jurkat cells treated without (control) or with 400 and 600 μM hydralazine (H-400 and H-600, respectively) for 24 hr. **C.** Mock-transfected (Jurkat/pCDNA), Bcl-2-overexpressing (Jurkat/Bcl-2) and Bcl-x_L_-overexpressing (Jurkat/Bcl-x_L_) Jurkat cells were treated without (control) or with 400 and 600 μM hydralazine (H-400 and H-600, respectively) for 48 hr. Sub-G1 cells were determined by flow cytometry. The inset figure shows the levels of Bcl-2, Bcl-x_L_ and β-actin in mock-transfected, Bcl-2- and Bcl-x_L_-overexpressing Jurkat cells, as determined by Western-blot. **D.** Mitochondrial membrane potential and ROS production were analyzed by flow cytometry in mock-tranfected, Bcl-2- and Bcl-x_L_-overexpressing Jurkat cells treated without (control) or with 600 μM hydralazine for 24 hr. Error bars in C show SEM from three independent experiments. ***p < 0.001.

Considering that caspase-9 is the initiator caspase responsible for the mitochondrial apoptosis, we analyzed several other features of this pathway in order to define the mechanism of hydralazine-induced apoptosis in leukemia T cell lines. Treatment of Jurkat cells with hydralazine resulted in generation of ROS and disruption of ΔΨm. Both events could be detected after 16 hr of incubation. In fact, the FACS profiles suggested that mitochondrial membrane depolarization preceded ROS accumulation, as all cells producing ROS showed low ΔΨm, whereas not all cells with depolarized mitochondria showed increased ROS production (Figure 2A).

The proapoptotic proteins Bax and Bak are essential for the initiation of mitochondrial dysfunction during apoptosis and their effects are antagonized by other members of the Bcl-2 family, such as Bcl-x_L_ and Bcl-2 itself. Since Jurkat cells are Bax-deficient, we analyzed Bak activation in response to hydralazine by using an active conformation-specific anti-Bak antibody. As shown in Figure 2B, a significant activation of Bak could be observed after 24 hr treatment. In addition, we studied the effect of hydralazine in Jurkat cells overexpressing Bcl-2 or Bcl-x_L_ [23, 26]. We observed that overexpression of these antiapoptotic proteins prevented hydralazine-induced cell death as compared with mock-transfected Jurkat cells (Figure 2C), thus confirming the involvement of mitochondria in this process. As expected, overexpression of Bcl-2 or Bcl-x_L_ also inhibited loss of ΔΨm and ROS accumulation in response to hydralazine (Figure 2D).

**Figure 3:**
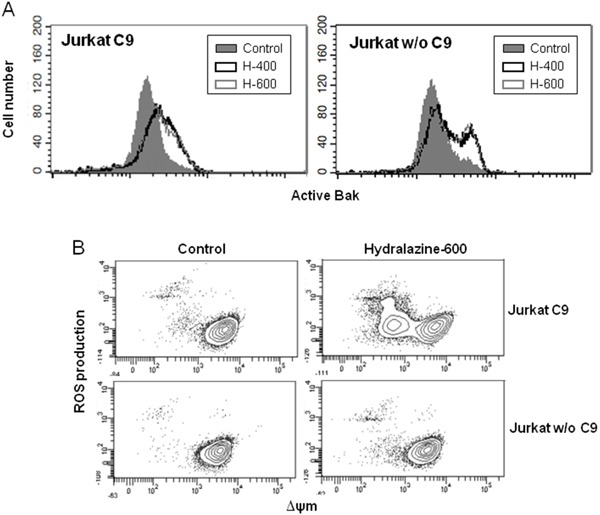
Induction of mitochondrial events by hydralazine in caspase-9 deficient cells **A.** Bak activation was analyzed by flow cytometry in caspase-9-deficient (Jurkat w/o C9) and caspase-9-reconstituted (Jurkat C9) Jurkat cells after treatment without (control) or with 400 and 600 μM hydralazine (H-400 and H-600, respectively) for 24 hr. **B.** Mitochondrial membrane potential and ROS production were determined by flow cytometry in caspase-9-deficient (Jurkat w/o C9) and caspase-9-reconstituted (Jurkat C9) Jurkat cells treated without (control) or with 600 μM hydralazine for 24 hr.

To further delineate the intrinsic apoptotic pathway triggered by hydralazine, we analyzed the activation of mitochondrial events in caspase-9-deficient Jurkat cells. Bak activation was similar in both caspase-9-lacking and reconstituted Jurkat cells (Figure 3A). However, hydralazine failed to induce ROS production and disruption of ΔΨm in cells deficient in caspase-9 (Figure 3B).

**Figure 4:**
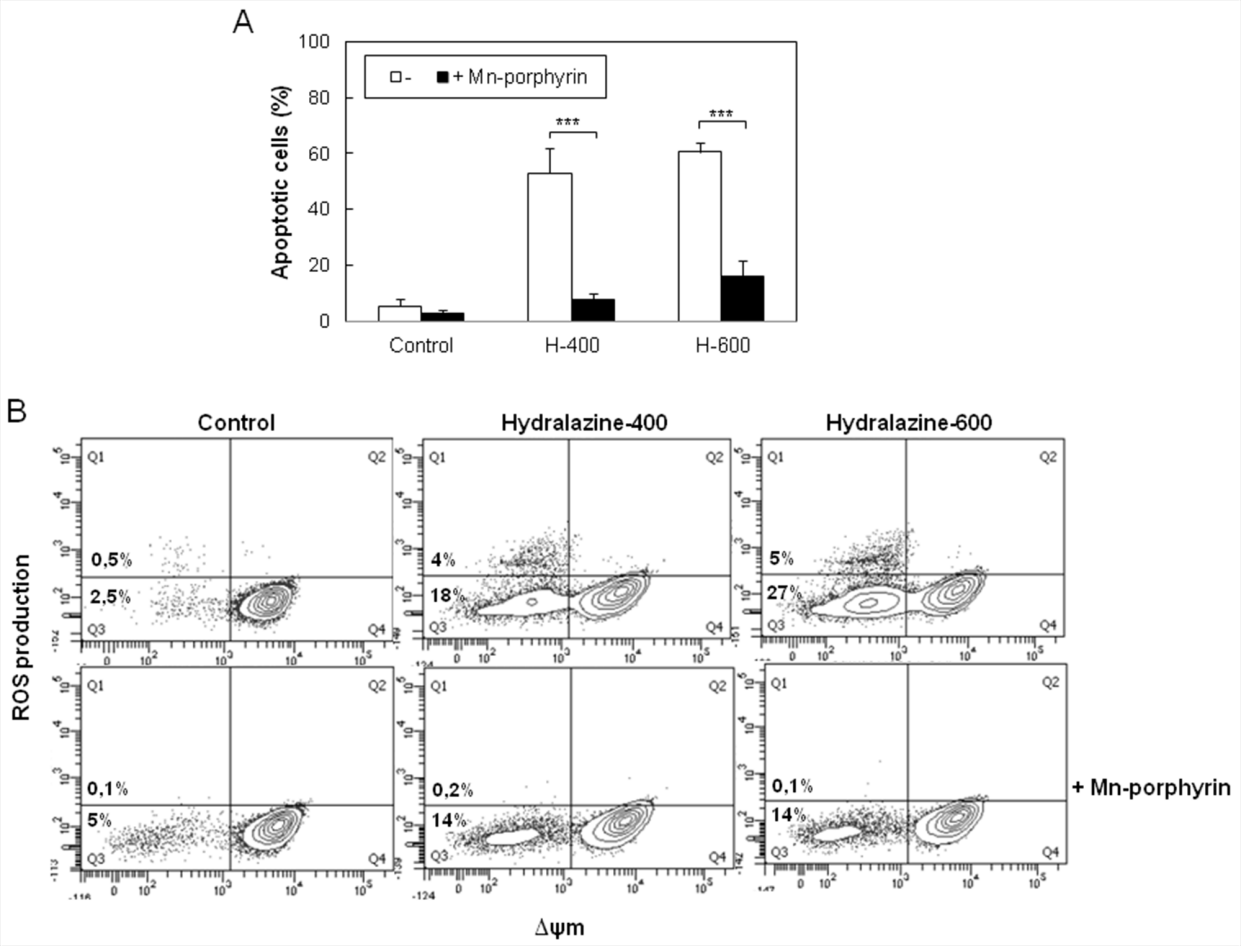
ROS scavenging prevents hydralazine-induced apoptosis in leukemic T cells **A.** Jurkat cells were preincubated for 24 hr in the absence or in the presence of 250 μM manganese porphyrin Mn(III)TMPyP and then treated without (control) or with 400 and 600 μM hydralazine (H-400 and H-600, respectively) for 48 hr. Hypodiploid apoptotic cells were determined by flow cytometry. Error bars show SEM from three independent experiments. ***p < 0.001. **B.** Mitochondrial membrane potential and ROS production were analyzed by flow cytometry in Jurkat cells treated for 24 hr as in A.

Accumulation of ROS results in oxidative damage to cell structures and molecules that lead to cell dysfunction and death [27]. To establish whether ROS generation was involved in the induction of apoptosis by hydralazine, we studied the effect of the cell-permeable superoxide dismutase (SOD) mimetic manganese-porphyrin, Mn(III)TMPyP. When Jurkat cells were pretreated with the manganese-porphyrin, a significant inhibition of hydralazine-mediated apoptosis was observed (Figure 4A). We confirmed the ability of the SOD mimetic to inhibit ROS production in response to hydralazine (Figure 4B). Strikingly, inhibition of ROS accumulation partially prevented the dissipation of ΔΨm (Figure 4B), even though mitochondria depolarization seemed to precede ROS production.

### Hydralazine induces DNA damage in leukemic T cells

**Figure 5:**
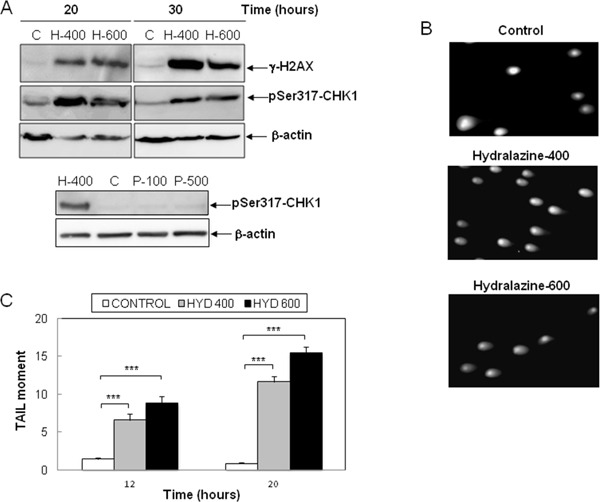
Induction of DNA damage by hydralazine in leukemic T cells **A.** Expression of γ-H2AX and phospho-Chk1 (Ser317) was analyzed by Western blot in Jurkat cells in response to treatment without (C) or with 400 and 600 μM hydralazine (H-400 and H-600, respectively) for the indicated times (upper panel). Lower panel shows the expression of phospho-Chk1 (Ser317) in Jurktat cells incubated without (C) or with 100 and 500 μM procainamide (P-100 and P-500, respectively) for 30 hr and hydralazine (H-400) was used as a positive control. β-Actin was used as loading control. **B.** and **C.** DNA strand breaks were analyzed by comet assay in Jurkat cells treated for 12 and 20 hr with 400 and 600 μM hydralazine. B. Images of cells with comet tails after 20 hr treatment. C. The comet tail moments were scored from at least 60 cells per sample (mean ± SEM). ***p < 0.001.

Previous reports have described the induction of DNA damage by nucleoside analogs DNMT inhibitors [23, 28]. Hence, as an early marker of DNA double-strand breaks, we analyzed the phosphorylation of the histone H2AX (γ-H2AX) in response to hydralazine. As shown in Figure 5A, high levels of γ-H2Ax were detected after 20 hr of treatment. We also determined the phosphorylation of Chk-1, a substrate of the DNA damage response kinases ATM and ATR. The phosphorylated and hence active form of Chk-1 was observed at all time points analyzed after treatment with hydralazine (Figure 5A). For comparison, we analyzed Chk1 phosphorylation in response to procainamide and observed that this drug did not induce DNA damage (Figure 5A, lower panel). We confirmed by comet assay the ability of hydralazine to induce DNA damage. Comet tails indicative of DNA strand breaks were clearly detected in cells treated with hydralazine (Figure 5B). Quantification of the tail moment in time-course experiments demonstrated that cells with significant DNA damage were already evident after 12 hr of hydralazine treatment (Figure 5C).

## DISCUSSION

The smooth muscle relaxant drug hydralazine has been recently included within the group of non-nucleoside analogue DNMT inhibitors. Different studies have described the high-affinity interaction between hydralazine and DNMT that may lead to the inhibition of the enzyme activity [15, 17]. In addition, it has been reported that hydralazine acts by reducing the expression of DNMT1 and DNMT3a rather than directly inhibiting DNTM enzymatic activity [16]. Whatever the precise mechanism of hydralazine-induced demethylation is, it has been shown to reactivate the expression of tumor suppressor genes silenced by methylation as well as to induce cell cycle arrest and apoptosis in some tumor cells [24, 25, 29].

In Jurkat leukemic T-cells, previous studies have reported that hydralazine decreases DNMT expression and activity and inhibits DNA methylation [16, 30]. Now, we show, for the first time, that hydralazine induces apoptosis in Jurkat and other leukemia T cell lines. Since aberrant methylation of several genes has been associated with the pathogenesis of T-cell acute lymphoblastic leukemia and the response to treatment of patients [31-33], our results support the possible use of hydralazine as a therapeutic strategy against this hematologic malignancy. We demonstrate that hydralazine induces caspase-dependent apoptosis in leukemic T cells at doses that show no significant toxicity for normal lymphocytes. Specifically, we show the activation of caspases-3 and -8 in response to hydralazine as well as the inhibition of apoptosis by the general caspase inhibitor Q-VD-OPh. In agreement with our data, previous studies suggested a cytotoxic activity of hydralazine in cervical, breast and prostate cancer cells [25, 34-36]. Nonetheless, our results are the first to describe the signaling pathway of hydralazine-induced apoptosis.

We have recently reported that the nucleoside analogues decitabine and zebularine induce mitochondria-mediated apoptosis in leukemic T cells [23]. Similarly, we have now observed that hydralazine induces Bak activation and loss of mitochondrial membrane potential followed by ROS production, which are all events associated with the mitochondrial apoptotic pathway. Moreover, Jurkat cells deficient for caspase-9, the upstream caspase of the intrinsic pathway, are resistant to hydralazine-induced apoptosis. Likewise, hydralazine does not trigger apoptosis in Jurkat cells overexpressing either Bcl-2 or Bcl-x_L_. Actually, all mitochondrial events are prevented in cells overexpressing these antiapoptotic proteins. All these data confirm the role of mitochondria in the induction of apoptosis by hydralazine in leukemic T cells. In the absence of caspase-9, there was neither a loss of ΔΨm nor an accumulation of ROS in response to hydralazine. However, upstream Bak activation was similar in caspase-9-proficient and -deficient Jurkat cells. These findings indicate that hydralazine triggers the activation of proapoptotic Bcl-2 family proteins, such as Bak, thus leading to the permeabilization of the mitochondrial outer membrane and the following release of intermembrane space proteins that allow the activation of the initiator caspase-9. Subsequent activation of effector caspases must give rise to the disruption of mitochondrial function [26, 37]. The mitochondrial damage seems to be essential for the complete dismantling of the cell, as suggested by the inhibition of hydralazine-induced apoptosis when preventing ROS accumulation with a SOD mimetic. Similar results were reported for decitabine and zebularine [23].

Our data suggest that hydralazine may activate an apoptotic pathway similarly to that triggered by nucleoside analogues, which is also comparable to that induced by genotoxic stress [26]. Moreover, similarly to genotoxic and nucleoside analogs drugs, hydralazine induces DNA damage in leukemic T cells, as determined by comet assay and activation of a DNA damage response. In line, induction of DNA damage by hydralazine has been recently reported in prostate cancer cells [36] and different authors have described the generation of radicals derived from the oxidation of hydralazine that may directly induce DNA damage [38, 39]. Regardless of the mechanism of DNA damage induction by hydralazine in leukemic T cells, this event must play an important role in apoptosis, as it is clearly evident after 12 hr treatment, concurrent with the activation of the apoptotic signaling pathway.

Comparing the effects of hydralazine with that of nucleoside analogs in leukemic T cells, minor differences are found [23]. First, the kinetic of DNA damage and apoptosis induction seems to be faster in response to hydralazine. In addition, inhibition of ROS accumulation partially prevents the dissipation of ΔΨm in response to hydralazine, even though mitochondria depolarization seems to precede ROS production, thus suggesting a positive feedback loop between both mitochondrial events that was not observed for nucleoside DNMT inhibitors. Interestingly, the leukemic T cell lines used in our studies lack functional p53. Other authors have reported the up-regulation of p53 in HeLa cells in response to hydralazine, although they did not demonstrate a direct relationship between p53 up-regulation and the antitumor effect of this drug [40]. Therefore, hydralazine may be considered as a novel therapeutic strategy that, similar to decitabine and zebularine and more efficiently than them, induces apoptosis by a p53-independent mechanism, at least in leukemic T cells.

Regarding the significance of the demethylating effect of hydralazine in the induction of apoptosis, a reduction of DNMT1 protein level and activity has been observed in ovarian cancer cells in response to treatment for one day with hydralazine in a range of doses from 150 to 600 μM [24]. However, the studies of Cornacchia et al. described the inhibition of DNA methylation in Jurkat cells upon treatment for more than five days with lower doses of hydralazine than those required to induce apoptosis in our study [30]. Here, we have shown that activation of apoptosis signaling and induction of DNA damage preceded depletion of DNMT1 in response to high doses of hydralazine. The fast kinetics of apoptosis induction by hydralazine in leukemic T cells suggests that, at least in part, it may be independent of DNA demethylation. On the other hand, the ability of the antiarrhythmic procainamide to inhibit DNA methylation has been reported to be similar to that of hydralazine [29]. However, unlike hydralazine, we observed that procainamide does not induce apoptosis in any of the leukemic T cell lines used in the present study, even when used for three days (data not shown) at concentrations up to 500 μM, a dose that induces only a slight decrease of DNMT1 expression after 48 hr of treatment. Interestingly, procainamide did not activate a DNA damage response in leukemic T cells.

In agreement with previous reports describing the low toxicity of hydralazine in peripheral blood mononuclear cells and human umbilical vein endothelial cells [25, 41], we show that higher doses of hydralazine have a minimal effect on the viability of normal peripheral blood lymphocytes. Preclinical studies with hydralazine, mostly in combination with the histone deacetylase inhibitor valproic acid, have supported its safety, good tolerability as well as its anticancer efficacy. A treatment combination with hydralazine has not only shown promising results in myelodysplastic syndrome [20], but also seems to increase the efficacy of chemotherapy in breast and cervical cancer [19, 21], and improve the response to imatinib in patients with chronic myeloid leukemia refractory to this drug [42]. Hydralazine has proven to induce the expression of tumor suppressor genes without producing global DNA demethylation in patients with cervical cancer [43]. Furthermore, it has been suggested that treatment with hydralazine and valproic acid enhances the effects of immunotherapy in cancer by regulating the expression of certain molecules, such as HLA class-I, Fas and MIC-A/-B, in tumor cells [44, 45]. Therefore, hydralazine seems to be a promising option for cancer treatment.

## MATERIALS AND METHODS

### Reagents and antibodies

Hydralazine hydrochloride, procainamide, dihydroethidium (DHE) and mouse anti-β-actin monoclonal antibody were from Sigma-Aldrich (ST. Louis, MO). The fluorescent cationic lipophilic dye 3,3′-dihexyloxacarbocyanine iodide (DiOC_6_ (3)) and Alexa Fluor 488-labeled goat anti-mouse were obtained from Molecular Probes (Carlsbad, CA). Manganese-porphyrin Mn(III)TMPyP was from Cayman Chemical (Ann Arbor, MI). Q-VD-OPh, a wide-spectrum caspase inhibitor, and anti-human caspase-9 monoclonal antibody were from R&D Systems (Minneapolis, MN). Cytofix/cytoperm was from BD Biosciences (San José, CA). Anti-Bak (Ab-1) monoclonal antibody and anti-Chk1 (pSer317) rabbit polyclonal antibody were obtained from Calbiochem (Darmstadt, Gemany). Anti-phospho-histone H2AX (Ser139) monoclonal antibody was from Upstate/Millipore (Billerica, MA). Anti-human caspase-8 monoclonal antibody was purchased from Cell Diagnostica (Munster, Germany). Anti-human caspase-3 polyclonal antibody was obtained from Stressgen (Ann Arbor, MI). Mouse monoclonal DNMT1 antibody was from Abcam (Cambridge, UK).

### Cells and cell culture

The human leukemic T cell lines Jurkat, MOLT-4 and CEM-6 were kindly provided by Dr. Abelardo López-Rivas (CABIMER, Sevilla, Spain). Jurkat cells deficient in caspase-9 and caspase-9 reconstituted Jurkat cells have been previously reported [26]. Jurkat cells stably overexpressing Bcl-2 or Bcl-x_L_ were generously provided by Dr. Jacint Boix (Departamento de Ciencias Médicas Básicas, Universidad de Lleida, Spain). Blood samples were obtained from healthy donors by informed consent and collected into citrates tubes. Peripheral blood lymphocytes (PBL) were then isolated by Ficoll–Histopaque density gradient centrifugation (Sigma–Aldrich) and depletion of adherent monocytes by culture on plastic dishes for 1 hr at 37°C. All cell lines were maintained in RPMI 1640 medium with 10% fetal bovine serum (FBS), 1 μM l-glutamine, penicillin and streptomycin at 37°C in a humidified 5% CO_2_, 95% air incubator. Bcl-2- and Bcl-x_L_-overexpressing cells were maintained in culture medium with 1 mg/ml G418 sulfate (Sigma Chemical).

### Determination of apoptotic cells

Hypodiploid apoptotic cells were detected by flow cytometry according to published procedures [46]. Briefly, cells were washed with PBS, fixed in cold 70% ethanol and then stained with propidium iodide. Quantification of sub-G1 apoptotic cells was carried out in a FACScan cytometer using the Cell Quest software (BD Biosciences).

### Flow cytometric analysis of Bak activation

Cells (2.5 × 10^5^) were washed in PBS, fixed in cytofix/cytoperm for 20 min on ice and washed with PBS/0.05% saponine solution. Cells were then incubated with 1:50 anti-active Bak antibody in staining buffer (PBS containing 10% FBS) for 30 min at room temperature in the dark. After washing with PBS/saponine, cells were incubated in staining buffer with 0.2 μg Alexa Fluor 488-labeled goat anti-mouse for 30 min at room temperature in the dark. Cells were washed and analyze in a FACScalibur cytometer using the Cell Quest software.

### Determination of mitochondrial membrane potential and ROS production

Mitochondrial membrane potential (ΔΨm) and reactive oxygen species (ROS) generation were analyzed by flow cytometry. Briefly, cells (2.5 × 10^5^) were incubated in RPMI 1640 medium supplemented with 5% FBS and containing the oxidant-sensitive probe DHE (2 μM) and the fluorescent potentiometric dye DIOC_6_ (3) (10 nM) for 20 min at 37°C in the dark. Quantitative analysis was carried out in a FACSCanto cytometer with the Cell Quest software.

### Immunoblot detection of proteins

Total protein extracts were prepared by lysing cells in ice-cold lysis buffer (150 mM NaCl, 50 mM Tris-HCl and 1% NP-40) for 30 min at 4°C. Proteins were resolved on SDS-PAGE minigels and detected as reported previously [47].

### Comet assay

DNA damage was quantified using Comet Assay kit (R&D Systems) as described previously [23]. Cells were analyzed by fluorescence microscopy (LEICA). Sixty to eighty cells were evaluated in each sample using the Comet Assay Software Project (CASP). DNA damage was quantified by measuring the tail moment (TM) calculated as percentage of DNA in the tail × tail length.

### Statistical analysis

The data were analyzed with unpaired Student's t-tests (two-tailed) by using GraphPad Prism 4 for Windows. Values of p < 0.05 were considered significant.
